# Age-Related Changes of the Pineal Gland in Humans: A Digital Anatomo-Histological Morphometric Study on Autopsy Cases with Comparison to Predigital-Era Studies

**DOI:** 10.3390/medicina57040383

**Published:** 2021-04-15

**Authors:** Bogdan-Alexandru Gheban, Horațiu Alexandru Colosi, Ioana-Andreea Gheban-Rosca, Bogdan Pop, Ana-Maria Teodora Domșa, Carmen Georgiu, Dan Gheban, Doinița Crișan, Maria Crișan

**Affiliations:** 1 Department of Anatomic Pathology, Iuliu Hațieganu University of Medicine and Pharmacy, 400129 Cluj-Napoca, Romania; pop.bogdan21@gmail.com (B.P.); domsa_dora@yahoo.com (A.-M.T.D.); carmengeorgiu@hotmail.com (C.G.); dgheban@gmail.com (D.G.); doinitacrisan@gmail.com (D.C.); 2 Emergency Clinical County Hospital, 400129 Cluj-Napoca, Romania; mcrisan7@yahoo.com; 3 Department of Medical Informatics and Biostatistics, Iuliu Hațieganu University of Medicine and Pharmacy, 400129 Cluj-Napoca, Romania; 4 The Oncology Institute “Ion Chiricuţă”, 400015 Cluj-Napoca, Romania; andreeaioana.rosca@yahoo.com; 5 Children’s Emergency Clinical Hospital, 400000 Cluj-Napoca, Romania; 6 Department of Histology, Iuliu Hațieganu University of Medicine and Pharmacy, 400129 Cluj-Napoca, Romania

**Keywords:** pineal gland, calcifications, immunohistochemistry, morphometry, anatomy, histology, autopsy

## Abstract

*Background and objectives:* The pineal gland is a photoneuroendocrine organ in the midline of the brain, responsible primarily for melatonin synthesis. It is composed mainly of pinealocytes and glial tissue. This study examined human postmortem pineal glands to microscopically assess age-related changes using digital techniques, and offers a perspective on evolutionary tendencies compared to the past. *Materials and Methods:* A retrospective autopsy study has been performed on 72 pediatric and adult autopsy cases. The glands have been processed for histological analysis and immunohistochemical staining with glial fibrillary acidic protein (GFAP). Slides were assessed under polarized light and digitally scanned. Morphometric data were obtained using CaseViewer and ImageJ. *Results:* Thirty-three females and 39 males were included in the study, grouped under three age groups: 0–25, 46–65, and 66–96 years of age. The peak gland volume was found within the 46–65 age group, the overall mean volume was 519 mm^3^, the main architectural types were lobular and insular, and the mean percentage of pineal calcification was 15% of the gland, peaking within the 66–96 age group, with a predominantly globular shape. Glial cysts were found in 20.8% of cases. The intensity of GFAP stain was maximal in the pediatric age group, but the extent of glial tissue was much larger in elderly patients. Discussion: The degenerative process of the pineal gland can be quantified by measuring normal parenchyma, calcifications, glial tissue, and glial cysts. Morphometric differences have been observed and compared to a similar studies performed in the published literature. The current study, unfortunately, lacks a 26–45 age group. Digital techniques seemed to offer a more exact analysis, but returned similar results to studies performed over 40 years ago, therefore offering important information on evolutionary tendencies. *Conclusions:* Increase in glial tissue, calcifications, and glial cysts have a defining role as age-related changes in the pineal gland.

## 1. Introduction

The pineal gland is a photoneuroendocrine organ that is part of the brain with an intricate role in circadian rhythm regulation and hormone synthesis [1]. The main hormone synthesized is melatonin, regulated by light stimuli [2,3]. In addition to melatonin, synthesis of serotonin and N,N-dimethyl-tryptamine has also been demonstrated in mammals [4]. Anatomically, the pineal gland is part of the epithalamus and is connected with the two recesses of the third ventricle, engulfed within the choroidal plexus [5]. It is situated on the sagittal line of the brain, has a pine cone shape and a red-greyish color, measures 5–9 mm in length and 2–5 mm in width, and weighs 100–180 mg [2]. There are numerous nerve fibers offering input to the pineal gland, one from the intrapineal ganglion, peri pineal ganglia, nervii conarii, and commissural peduncles, and numerous smaller ones, both myelinated and unmyelinated, most leading to the pretectal area. [6]

Histologically the pineal gland is composed of follicles and chords of pinealocytes residing within glial tissue [7]. Some glands are arranged in lobules, while others are in islands, with variable amounts of glial and connective tissue [8]. Pinealocytes have a poorly delineated cytoplasm, with numerous appendages. The nucleus contains neuroendocrine vesicles that are positive for the immunohistochemical marker synaptophysin [8,9]. Pinealocytes also stain positive for neuron-specific enolase, while the glial tissue stains positive for glial fibrillary acidic protein (GFAP) [5,10]. Xu Zang has demonstrated different immunohistochemical patterns for pineal astrocytes depending on species, and in humans the GFAP antibody ties to a moderate population of astrocytes, and especially the glial processes that form a meshwork in the entire gland [11].

Calcifications or “corpora arenacea” are often found in the pineal gland, with a globular or concentric lamellar shape [6]. The incidence of these calcifications is increased over the age of 30 [7]. Their composition is shown to be made up mostly of calcium, carbon, and oxygen. The crystals present either a cubical, cylindrical, or hexagonal shape in spectroscopic studies [12]. There are numerous theories regarding the mechanisms for pineal calcification formation, including the role of tryptase-containing mast cells, extrusion of polypeptides in the extracellular space with an active transfer of calcium, and the possibility of osteoblast-like transformation of local stem cells, known to cause germinal cell tumors within the pineal gland [13].

Glial cysts are also a common discovery in pineal glands; according to the literature they have an estimated prevalence of 25–40%, mostly residing within glial areas [8,14]. The cysts have been detected in adults, while their presence in children has been estimated to be around 3% of investigated cases [15]. The cysts are composed of a glial layer, pineal layer, and Rosenthal fibers [10]. The content of the cysts is cerebrospinal fluid (CSF), often leading to confusion in magnetic resonance imaging (MRI) due to the nature of the CSF hyperintensity, requiring differential diagnosis with atypical cysts, germ-cell tumors, and pineal parenchymal tumors [16].

The activity of the pineal gland is tied to its volume and amount of active parenchyma. As the parenchyma gradually is replaced by calcifications and glial tissue and undergoes cystic degeneration, the secretory activity of the gland diminishes [9]. To the best of our knowledge, there is currently very limited information available on age-related morphological changes of the gland, such as quantification of the calcifications, the degree of glial tissue replacing the glandular parenchyma, or cystic degeneration of the gland. Earlier studies have been done using less-objective morphometrical methods. Papasozomenos demonstrated in 1983 that there was a more pronounced astrocytic differentiation within the parenchyma of the organ in elderly patients [17].

The purpose of this study is to provide digitally acquired anatomical and histological morphometric data from human pineal glands, examined postmortem, using hematoxylin and eosin (H&E) and immunohistochemical stains, assessing the degree of calcification, particularities of these calcifications, volume, glial tissue, and presence of cysts in relation to age, gender, and certain clinical characteristics (obesity, type 2 diabetes mellitus, dementia, hypertension) of both pediatric and adult patients.

## 2. Materials and Methods

### 2.1. Case Selection

A total of 112 pineal glands were harvested from autopsies performed between January 2017 and March 2019, within the Emergency Clinical County Hospital and the Children’s Clinical Hospital of Cluj-Napoca, Romania, and were assessed for inclusion in the study. Forty glands were considered unsuitable for the analysis due to hemorrhagic infarctions of the brain with partial or total necrosis of the pineal gland, advanced autolysis, agenesia, or tumoral metastasis to this location. In total, 72 pineal glands were included in this retrospective study. The harvesting procedure included the choroidal plexus and the habenular area of the third ventricle recesses.

The study was conducted following the Declaration of Helsinki and its protocol was approved by the Ethics Committee of the Iuliu Hațieganu University of Medicine and Pharmacy Cluj-Napoca (278/5 July 2018). Anthropometric, clinical, histological, and morphometric data were collected anonymously.

### 2.2. Specimen Processing and Staining

Gross inspection and measurements on the x/y/z axes were performed. The volume of the gland (mm^3^) was calculated using the X, Y, Z measurements obtained macroscopically after harvesting, and before fixation, by applying the mathematical formula for an ellipsoid volume: 4/3*PI*X*Y*Z. The choroidal plexus and the habenular tissue were not included in the volume measurements.

The specimens were routinely fixed in 10% neutral buffered formalin, sectioned axially in half, processed, and then embedded in paraffin. The paraffin block was sectioned at 0.2 µm from the axial cut performed macroscopically, thus ensuring the median surface of analysis. Slides were manually stained using complementary median sections for hematoxylin and eosin and the immunohistochemical stain: glial-fibrillary acidic protein (RRID:Addgene_55051) 7 mL, clone GA5 by Leica Biosystems Inc (Buffalo Grove, IL, USA), using Detection Kit Immunohistochemistry and bond TM wash solution 10x concentrated, by Leica Biosystems Inc. The immunohistochemical staining procedure included placement of the section on a silanized slide, xylene deparaffination, and three successive alcohol baths followed by two successive distilled-water cleansings. The slide was washed in PBS, demasked, cooled, washed in PBS again, subjected to a peroxidase block using H_2_O_2_, washed in PBS again, then subjected to an unspecific protein reaction block and an hour of incubation of primary antibodies in a wet chamber. Another PBS wash followed, with post primary block sensibilization, PBS wash, secondary antibody incubation for 30 min, PBS wash, and 3,3′-Diaminobenzidine (DAB) incubation. Finally, each slide was washed with distilled water, counter-colored with Hematoxylin, cleansed, washed, inserted in alcohol and xylene for clarification, and mounted.

### 2.3. Slide Analysis

The slides were digitally scanned at 40x resolution using the 3DHISTECH PANNORAMIC SCAN II (Budapest, Hungary). The morphometric analysis was performed using the 3DHISTECH software CaseViewer (Budapest, Hungary) (CaseViewer, RRID:SCR_017654), Adobe Photoshop CC 2019 (San Jose, CA, USA) (Adobe Photoshop, RRID:SCR_014199), and ImageJ/Fiji (LOCI, University of Wisconsin, USA) (ImageJ, RRID:SCR_003070). All images used in the analysis were set using the same optical parameters, image parameters, scan settings, and hardware version, with a slide pixel dimension of 112640 × 243200 and over 9000 scanned fields of view.

A Leica DM2500 microscope (Buffalo Grove, IL, USA) with a polarized light plug-in was used for the analysis of calcifications of the H&E slides. Analysis of the IHC slides was performed using a staining score, also used by Fedchenko and Reifenrath in 2014 [16] as the immunoreactive score or IRS, combining a score of A (percentage of positive cells) and B (intensity of staining), obtaining the final IRS score (A × B): 0–12.

**Figure 1:**
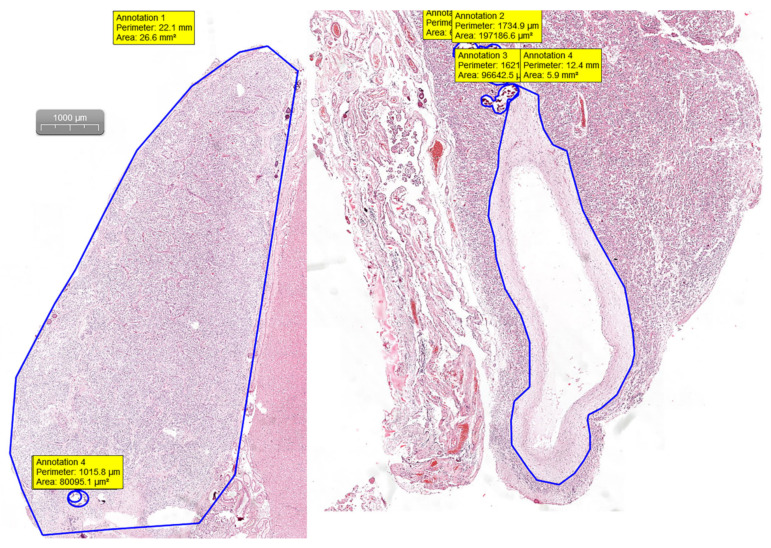
H&E digital slides used for measuring the perimeter and area using the “draw closed polygon” tool in CaseViewer by 3DHISTECH (Budapest, Hungary)**.**

Pineal gland measurements were performed on H&E digital slides. Slides were visualized and analyzed using the CaseViewer software by 3DHISTECH (Budapest, Hungary). By measuring the area and the perimeter of the section and the area and perimeter of the calcifications and cystic changes, we were able to assess the percentage of the replaced glandular parenchyma (Figure 1).

**Figure 2:**
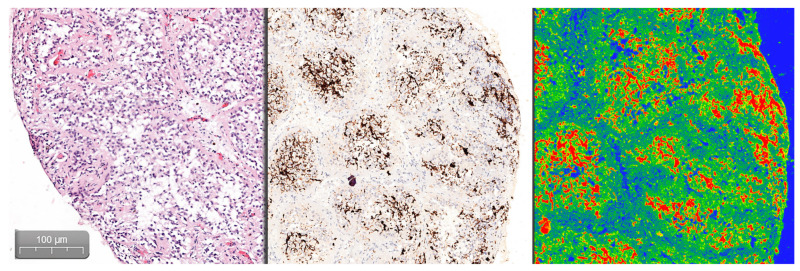
Comparative view of the H&E, GFAP, and gradient-map mode of the GFAP digital slide as seen in CaseViewer by 3DHISTECH (Budapest, Hungary).

**Figure 3:**
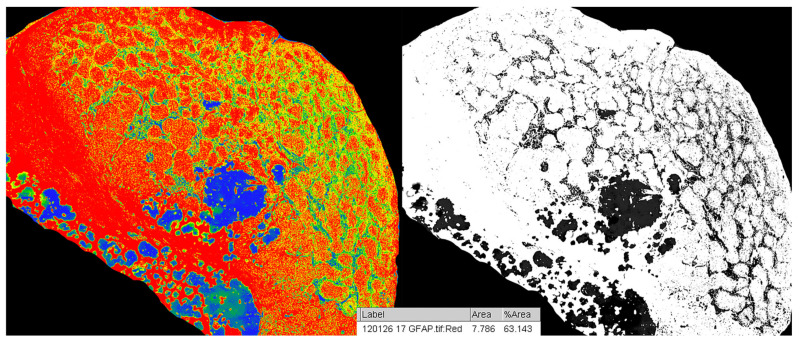
Analysis and area-percentage calculation of GFAP stain in pineal gland and greyscale with red threshold of 100–250 using ImageJ software.

**Figure 4:**
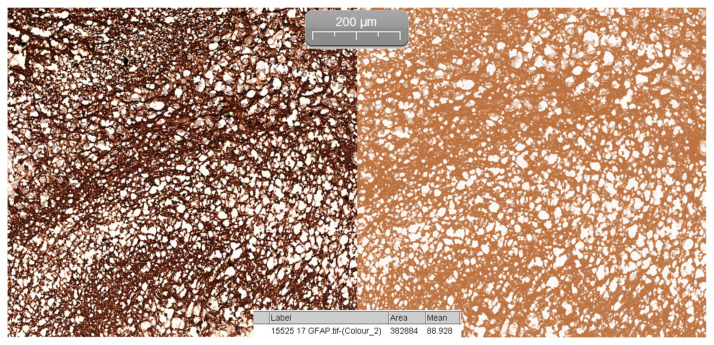
Color deconvolution of H DAB and calculation of mean grey value using ImageJ software.

Quantifying the percentage of GFAP positive tissue was done using the CaseViewer software by 3DHISTECH, using the Gradient Map Visualization plug-in (Figure 2). We obtained high-definition images that were cropped to include only the pineal gland, without nearby central nervous tissue or choroidal plexus. The images were analyzed using the ImageJ/Fiji software, with an image threshold for red of 100–250. The selected region of interest (ROI) was the pineal parenchyma, and the analyzed measurements were area and area fraction with threshold limit, thereby obtaining the percentage of glial tissue (Figure 3). The intensity of the immunohistochemical staining was quantified by using the digital IHC slides, viewed in the CaseViewer software, converted to high-definition images that were used in ImageJ/Fiji. After using the color-deconvolution tool, we established a region of interest within the best field of DAB pigment. We extracted a mean gray value that we used to calculate the optical density (OD) using the formula: OD = log (max intensity/mean intensity, with a maximum intensity of 255 for 8-bit images) (Figure 4).

### 2.4. Statistical Analysis

Kolmogorov–Smirnov and Shapiro–Wilk tests, as well as Q–Q plots, were used to assess the normality of quantitative variables. Continuous data exhibiting normal distribution were described as mean and standard deviation (SD), while continuous data exhibiting non-normal distribution were described as the median and interquartile range (IQR). Nominal variables were described using relative frequencies (percentages). Multiple comparisons of non-normally distributed variables were performed by using the Kruskal–Wallis method. Differences between investigated groups were evaluated with the aid of parametric tests (Student’s t-test and ANOVA) for continuous variables with normal distribution, and nonparametric tests (Mann–Whitney U test, Wilcoxon) for continuous variables with non-normal distribution. Post hoc tests with common corrections (Bonferroni, Tukey, Tamhane) were applied to investigate pairwise differences between the investigated groups. For proportions, Fisher’s test or Pearson’s chi-square test were applied, based on the appropriate counts of the expected frequencies tables. Full factorial general linear models (GLM) were used for the appraisal of possible correlations and interactions between investigated variables of interest. Backward stepwise logistic regression models were used to model the possible influence of binomial predictors on the presence of glial cysts. All tests were considered significant at *p*-values < 0.05. Statistical description and analyses were conducted using IBM SPSS Statistics 25 (Armonk, NY, USA) (IBM SPSS Statistics, RRID:SCR_019096) and Microsoft Excel 2016 (Redmond, WA, USA) (Microsoft Excel, RRID:SCR_016137).

## 3. Results

Thirty-three females and 39 males were included in the study. Six were 0–25 years of age, 21 were 46–65 years, and 45 were 66–96 years old. (Table 1).

The measured volumes of the pineal glands showed that the 46–65 age group had a mean volume 5x higher than the 0–25 age group (*p* = 0.003—Mann–Whitney test). The volume showed only a slight decrease in the 66–96 age group, compared to the 46–65 age group (*p* = 0.051—Mann–Whitney test). There was no significant volume difference between males and females (*p* = 0.647—Mann–Whitney test).

The architecture type of the pineal gland was significantly influenced by age group (*p* = 0.01—Fisher’s exact test). The histological pattern of the 0–25 age group was predominantly organized in sheets (50%), followed by mixed (33.3%) and insular pattern (16.7%). The 46–65 age group exhibited a mostly lobular structure (47.6%), followed by islands (42.9%) and mixed structures (9.5%), while the 66–96 age group had a predominantly insular architecture (42.2%), followed by lobular (37.8%), sheets (13.3%), and mixed (6.7%) architectural types.

The architectural type was also significantly influenced by gender (*p* = 0.039—Fisher’s exact test). The insular and lobular architecture was present in both genders, with males exhibiting a predominantly insular pattern (41%), and females a predominantly lobular pattern (42.4%). The mixed form was present only in males.

The percentage of calcified pineal parenchyma was 0% in the 0–25 age group, significantly different from the 14% in the 46–65 age group and the 15% in the 66–96 age group (*p* < 0.001—Mann–Whitney test). The observed calcifications had a predominantly globular shape (48.6% of cases), with the rest exhibiting a mix between concentric lamellar and globular or purely concentric lamellar shapes. The calcification form was significantly linked to the age group (*p* = 0.001—Fisher’s exact test). There was no statistically significant difference between males and females regarding the percentage of calcified parenchyma (*p* = 0.091—Mann–Whitney test).

The number of extrapineal calcifications (localized within the choroid plexus and surrounding the pineal gland) had a peak frequency in the 46–65 age group, after which the number slowly decreased in the 66–96 age group. Both these age groups exhibited a significantly higher number of extrapineal calcifications compared to the 0–25 age group, in which such calcifications were absent (*p* < 0.05—Mann–Whitney test). Only nine cases presented calcifications within the habenular glial tissue, with a pure globular shape.

**Figure 5:**
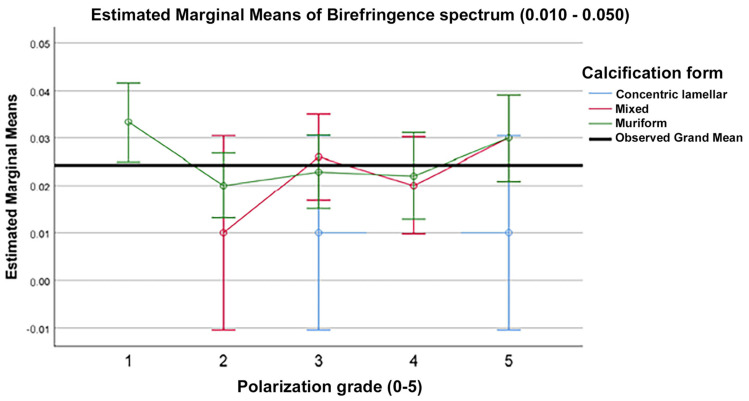
Estimated marginal means of the birefringence spectrum based on calcification form, polarization grade, and their interaction.

Polarized-light microscopic analysis of the parenchymal, choroidal, and habenular calcifications revealed that 87.5% of calcifications polarized. Polarization was found to be linked to the calcification form, with a significantly lower proportion of polarization occurring in concentric lamellar forms compared to globular or mixt calcifications (*p* = 0.019—Fisher’s exact test). Correlations that could help predict the birefringence spectrum based on polarization grade, calcification form, and their interaction were not found when using a full factorial GLM (Figure 5).

Based on logistic regression models, the presence of glial cysts in the pineal gland was not significantly linked with clinical characteristics such as obesity, type 2 diabetes mellitus, dementia, or hypertension.

The IRS score of the GFAP immunohistochemical stain had a peak value in the 0–25 age group, with its lowest value in the 46–65 age group, and slightly increasing in the 66–96 age group; however, no statistically significant difference between the three age groups could be proven in this study (*p* = 0.104—Kruskal–Wallis test). No statistically significant difference was found between males and females concerning this score (*p* = 0.787—Mann–Whitney test).

The presence of glial cysts within the pineal parenchyma was found to be 1 out of 6 (16.7%) within the 0–25 age group, 7 out of 21 (33.3%) within the 46–65 age group, and 7 out of 45 (15.6%) within the 66–96 age group. A pineal cyst was found in 20.8% of the total number of investigated cases, but the presence of a glial cyst could not be linked to a certain age group (*p* = 0.22—Fisher’s exact test).

## 4. Discussion

Our study focused on human pineal gland morphology using a modern, accurate approach allowed by digital techniques. It concerned three major age groups and was performed on 72 pineal glands harvested from autopsies. Most histological studies published so far in the literature were performed on animal species, while the majority of human studies were performed using radiological techniques on live patients. The current study is one of the few autopsy studies that has been performed on human pineal glands using digital histological morphometric analysis. This study focused on morphological changes that could be age-related. The morphometric analysis focused on identifying degenerative changes that occur within the gland, and aimed to find a recognizable pattern in relation to age. Another aim of the study was to add an objective mathematical morphometric analysis, using digital techniques, to validate nondigital analysis done in the past and to solidify the current morphological characteristics the pineal gland has shown so far in the literature.

The study of degenerative processes occurring in the pineal gland might explain certain neurological and endocrine disorders that elderly patients exhibit without any other known cause, as calcifications and increased glial tissue decrease the normal secretory activity of the pineal gland, especially with respect to melatonin and serotonin, as also suggested by Sandyk, Kay, and Gomez [18,19]. Further studies should investigate aspects of senile dementia in connection with symptoms such as disorientation and insomnia, as theorized by Bayliss et al. as early as 1985 [20]. The complete absence of intracerebral hemorrhage in our study could not establish a link to calcifications as suggested by Kitkhuandee et al. [21].

This study also aimed to open a broader evaluation of the pineal gland, especially during forensic and routine autopsies, in patients with known endocrine, neurological, and psychiatric pathologies, which might offer scientific arguments to support the final diagnostic outcome of the autopsy. This idea also was proposed by Kurtulus et al. after analyzing a possible role of pineal degeneration in suicide cases [22]. The increased staining intensity of GFAP can also be a valuable marker to evaluate possible post-traumatic events, as indicated by Neri et al., suggesting more attention should be paid to the pineal gland during routine forensic autopsies [23].

The mean volume of the pineal gland found in our study, was consistent with the findings of Golan et al. in terms of the 46–65 age group exhibiting the largest volume [24]. Our study found different volume values than the ones presented by Sigurdardottir et al., which were found in a cohort of Icelandic men, possibly confirming the role of light exposure and circadian rhythm on melatonin synthesis, and even on morphological changes of the glandular parenchyma itself [25]. Our study has found no significant differences between genders, with results being similar to the MRI study of Sun B et al. [26], and major differences being found primarily between age groups, contrary to the findings of Qing Han et al. in an MRI study [27]. The different approaches to morphometry could be a possible explanation for these differences; therefore, broader studies need to be planned and performed, for instance, to compare the accuracy of imaging techniques such as MRI with macroscopic and microscopic examinations.

The histological architecture documented in our study showed the same main types described, the same age variations by Koshy et al. and Tapp, and Zang et al., as well as the presence of glial tissue and sparse connective tissue described by nonimmunohistochemical morphometric studies [7,8,11,28].

Our study found an increased percentage of calcified parenchyma as age progressed, with an apparent major increase from 0 and 14%. Mrvelj and Womble demonstrated that a fluoride-free diet had a significant effect on pineal parenchymal growth, as there was increased pineocytic cellularity in rats without any fluoride intake. This could have important public-health implications, and the calcifications described need to have a more profound analysis to understand the role of fluoride in the genesis of parenchymal calcifications, as well as choroidal plexus metastatic calcifications [29]. Older studies suggest pineocytes have more calcium accumulations as their number is increased, and acervuli contain mostly calcium and phosphorus [30]. There is a need for further studies to assess the risk or possible causal factors of these calcifications, whether they are environmental, dietary, or stress-related. Polarized-light microscopy can be used to assess the nature of calcifications found within the pineal gland, as inferences may be drawn with inorganic studies performed on similar types of calcification. Further studies should be done using mass spectrometry or other biophysical evaluations of the composition of these calcifications.

Our findings coincide with the globular and concentric lamellar shapes of the intra-parenchymatous calcifications described by Koshy [7] and Baconnier et al. [12], as well as with the peak incidence of calcifications being situated in the middle-aged group, as stated by Tapp [8] and Whitehead et al. [15]. The lack of calcifications found in our study in the 0–25 age group differed from the findings of Doyle and Anderson’s CT study [31], but both our studies support the lack of difference in the number of calcifications between males and females. Extrapineal calcifications of the choroid plexus exhibited comparable frequencies in our study and in the one performed by Baconnier et al. [12].

The presence of pineal cysts in our study was three times lower than that presented by Sigurdardottir et al., while being 15 times higher than the Belgian MRI study by Golzarian et al., and the presence of calcifications was 6% smaller in our study compared to the Icelandic one, suggesting that cystic changes in the pineal gland might be influenced by the different seasonal-light exposure the Icelandic population receives naturally, compared to the Eastern European seasonal natural-light exposure [25,32].

Furthermore, the incidence, histological structure, and size of pineal cysts were very similar to the findings of Barboriak et al., Taraszewska, Wurtman, Moskowitz, Tapp, Pu, Milroy, Joo-Young, and Fain et al., keeping in mind the size differences that occur in MRI studies compared to histologic ones. None of the patients in our study presented symptoms suggestive of symptomatic glial cysts, nor was the size of any of the cysts in our study big enough to be considered a high-risk cyst [8,10,14,23,33,34,35,36].

The immunoreactivity score (IRS) used for the GFAP IHC stain could be used as a valid parameter in evaluating the extent of gliosis present within the pineal gland, as it assessed both the intensity of stain and the percentage of parenchyma that was positive for the antibody. The results of the current study showed an increased IRS score in the pediatric age group due to the intensity scores of the stain and a large number of glial cells present; however, the density of glial tissue was reduced in its expansion. Within the middle-aged group, there was a lower intensity and a moderate extension of the glial tissue, while in the higher-aged group, our study found the largest expansion of glial tissue replacing the functional parenchyma, with a greatly reduced nuclear density of astrocytes in comparison with the pediatric age group, a process that also favors the formation of glial cysts. Due to this confusion, it is always best to keep in mind the separate values that make up the IRS score and not to use it as a standalone prediction marker for gliosis. Reactive gliosis adjacent to ruptured cysts can interfere with these results, but we had no such cases in our study. The different proportions of astrocytic processes and their role in modulating neurosecretory activity, as described by Zang et al., show that there is increased pineal secretory activity at a younger age, compared to the elder population of our study [11]. More morphometric studies are required to evaluate the proper secretory activity of the gland by using immunohistochemical markers for neuroendocrine cells and activity, such as synaptophysin and neuron-specific enolase.

The peripheric staining of the islands and lobules of pineal parenchyma highlights the presence of different astrocytic cells, as described by Erlich and Apuzzo [5], making this an important histological element of diagnosis [22], and thus allowing this information to be used in the differential diagnosis between normal parenchyma and well-differentiated pineocytoma. Our study regarding gliosis confirmed the findings of Tapp’s 1979 study on the histology of the pineal gland [8], and showed a pattern similar to the small human study done by Zang et al. in 1985 [11].

Sozos published a study in 1983 regarding the GFAP-staining cells of the human pineal gland, with very similar findings as our study, on a large set of pineal glands harvested during autopsies. Our study offers a digital morphometric and statistical analysis that validates the findings and consolidates the presence, role, and importance of glial fibrillary processes within the pineal gland from a young age, and the changes that occur late in life. [17]. GFAP-positive elements overlapped with the findings of a similar study conducted by Gomez et al. on cow pineal glands [19].

The use of digital slides and specific morphometric software has enabled a much more complex view of histology and histopathology, allowing much more exact measurements and the possibility to establish predictability of the growth pattern of tissue and the evolution of the disease. This aspect has been a fresh new perspective in the field of digital pathology. Histopathological image analysis has been reviewed and encouraged by Gurcan et al., and our study also highlighted the importance of digitizing the microscopic analysis [37].

The similarities found with older studies from the 1970s until the present, despite the different approaches to morphometry, showed that there are no evolutionary tendencies of degeneration within the pineal gland, and external factors such as climate change, pollution exposure, or the presence of microplastics do not influence the morphological changes of the pineal gland to such an observable degree. Further studies are required to link the existence of calcifications and a diet-linked habit. The most observable differences were found in the comparison of two distinct populations from Iceland and Romania in terms of sunlight exposure, circadian rhythms, and annual seasons. Further studies from different locations would offer more concrete information.

The main strength of this study is represented by the relatively large number of human pineal glands that were examined using digital slides and software for analysis. While a larger number of subjects would have been desirable in the younger age groups, there are currently very few studies that used human tissue, regardless of age group. The major limitation of this study was the complete absence of pineal glands from the 26–45 age group, due to the rarity of autopsies being performed on individuals in this age group within the pathology department where this study was performed. Our study did not weigh the glands to obtain a volume due to a lack of technical equipment for such purposes in our department.

The findings of our study pave the way for further research, not just histological, but biochemical and ultrastructural as well, thereby justifying the inclusion of the pineal gland as an organ that should be evaluated in the practice of endocrinologists, neurologists, psychiatrists, and pathologists.

## 5. Conclusions

The main age-related changes of the pineal gland include the replacement of the normal secretory parenchyma of the pineal gland with glial tissue, connective tissue, calcifications, and glial cysts. No significant gender-related differences were found. The architecture of the gland also had a changing pattern as age progressed. Choroid plexus calcifications, a form of metastatic calcifications, have a less important role in the overall calcification of the pineal gland, as parenchymatous calcifications have different morphological and polarization characteristics. The nature of these calcifications need to be further studied, with polarized light offering basic data to work with. There are differences in findings that have been published so far from different geographic locations regarding these degenerative changes, with the main differences possibly being related to seasonal light-exposure variations throughout the world. Further studies are needed to assess the clinical impact of these degenerative changes on the endocrine and nervous systems, with possible psychiatric, neurological, and endocrine disturbances. A digital approach consolidated many of the previous morphological studies of the pineal gland and brought new understandings, but raised more questions regarding the particularities of this organ.

## Figures and Tables

**Table 1:** Evaluated parameter results and standard deviation in relation to age groups

Evaluated Parameters	Age Groups ^(Standard Deviation)^		
	0–25 Years	46–65 Years	66–96 Years
Mean age (years)	4 ^(5.7)^	58 ^(5.1)^	79.5 ^(8.1)^
Mean gland volume (mm^3^)	112 ^(133.5)^	519 ^(357.2)^	379 ^(368.3)^
Mean pineal field (mm^2^)	12.5 ^(11.7)^	34.2 ^(18.2)^	27.5 ^(17)^
Mean GFAP intensity (0–10)	9 ^(0.6)^	6.8 ^(1.9)^	6.9 ^(2.4)^
Mean GFAP percentage (%)	46 ^(19.1)^	46 ^(12.5)^	48 ^(12.3)^
Mean GFAP IRS score	7.5 ^(7.5)^	5.3 ^(6)^	5.7 ^(2.3)^
Mean calcified parenchyma field (mm^2^)	0 ^(0)^	4.7 ^(5)^	4.6 ^(10.6)^
Mean calcified parenchyma percentage (%)	0 ^(0)^	14 ^(13.7)^	15 ^(21.4)^
Total number of choroid plexus calcifications	0 ^(0)^	6.5 ^(14.9)^	5.8 ^(9.3)^
Total number of glial cysts found/Total number of cases	1/6	7/21	7/45
Mean area of parenchyma replaced by cystic degeneration (mm^2^)	0.4 ^(1)^	1.6 ^(2.9)^	1.5 ^(6)^
Mean percentage of parenchyma replaced by cystic degeneration (%)	1.2 ^(3.1)^	3.7 ^(6.6)^	2.9 ^(9.8)^

## Data Availability

No data was reported.

## References

[1] Stehle J.H., Saade A., Rawashdeh O., Ackermann K., Jilg A., Sebestény T. (2011). A survey of molecular details in the human pineal gland in the light of phylogeny, structure, function and chronobiological diseases. J. Pineal Res..

[2] Macchi M.M., Bruce J.N. (2004). Human pineal physiology, and functional significance of melatonin. Front. Neuroendocrinol..

[3] Ostrin L.A. (2019). Ocular and systemic melatonin and the influence of light exposure. Clin. Exp. Optom..

[4] Barker S.A., Borjigin J., Lomnicka I., Strassman R. (2013). LC/MS/MS analysis of the endogenous dimethyltryptamine hallucinogens, their precursors, and major metabolites in rat pineal gland microdialysate. Biomed. Chromatogr..

[5] Erlich S.S., Apuzzo M.L. (1985). The pineal gland: Anatomy, physiology, and clinical significance. J. Neurosurg..

[6] Sparks D.L. (1998). Anatomy of a new paired tract of the pineal gland in humans. Neurosci. Lett..

[7] Koshy S., Vettivel S.K. (2001). Varying appearances of calcification in human pineal gland: A light microscopic study. J. Anat. Soc. India.

[8] Tapp E. (1979). The histology and pathology of the human pineal gland. Prog. Brain Res..

[9] Nölte I., Lütkhoff A.T., Stuck B.A., Lemmer B., Schredl M., Findeisen P., Groden C. (2009). Pineal volume and circadian melatonin profile in healthy volunteers: An interdisciplinary approach. J. Magn. Reson. Imaging.

[10] Taraszewska A., Matyja E., Koszewski W., Zaczyński A., Bardadin K., Czernicki Z. (2008). Asymptomatic and symptomatic glial cysts of the pineal gland. Folia Neuropathol..

[11] Zang X., Nilaver G., Stein B.M., Fetell M.R., Duffy P.E. (1985). Immunocytochemistry of pineal astrocytes: Species differences and functional implications. J. Neuropathol. Exp. Neurol..

[12] Baconnier S., Lang S.B., Polomska M., Hilczer B., Berkovic G., Meshulam G. (2002). Calcite microcrystals in the pineal gland of the human brain: First physical and chemical studies. Bioelectromagnetics.

[13] Tan D.X., Xu B., Zhou X., Reiter R.J. (2018). Associated Health Consequences and Rejuvenation of the Pineal Gland. Molecules.

[14] Wurtman R.J., Moskowitz M.A. (1977). The pineal organ (Second of two parts). N. Engl. J. Med..

[15] Whitehead M.T., Oh C., Raju A., Choudhri A.F. (2015). Physiologic pineal region, choroid plexus, and dural calcifications in the first decade of life. AJNR Am. J. Neuroradiol..

[16] Fedchenko N., Reifenrath J. (2014). Different approaches for interpretation and reporting of immunohistochemistry analysis results in the bone tissue—A review. Diagn. Pathol..

[17] Papasozomenos S.C. (1983). Glial fibrillary acidic (GFA) protein-containing cells in the human pineal gland. J. Neuropathol. Exp. Neurol..

[18] Sandyk R., Kay S.R. (1991). The relationship of pineal calcification and melatonin secretion to the pathophysiology of tardive dyskinesia and Tourette’s syndrome. Int. J. Neurosci..

[19] Gómez Esteban M.B., Muñoz M.I., Carbajo S., Carvajal J.C., Alvarez-Morujo A.J., Barragán L.M. (2008). Pineal gliosis and gland ageing. The possible role of the glia in the transfer of melatonin from pinealocytes to the blood and cerebrospinal fluid. Eur. J. Anat..

[20] Bayliss C.R., Bishop N.L., Fowler R.C. (1985). Pineal gland calcification and defective sense of direction. Br. Med. J..

[21] Kitkhuandee A., Sawanyawisuth K., Johns J., Kanpittaya J., Tuntapakul S., Johns N.P. (2014). Pineal calcification is a novel risk factor for symptomatic intracerebral hemorrhage. Clin. Neurol. Neurosurg..

[22] Kurtulus Dereli A., Demırci G.N., Dodurga Y., Özbal S., Cankurt U., Boz B., Adiguzel E., Acar K. (2018). Evaluation of human pineal gland acetylserotonin O-methyltransferase immunoreactivity in suicide: A preliminary study. Med. Sci. Law.

[23] Milroy C.M., Smith C.L. (1996). Sudden death due to a glial cyst of the pineal gland. J. Clin. Pathol..

[24] Golan J., Torres K., Staśkiewicz G.J., Opielak G., Maciejewski R. (2002). Morphometric parameters of the human pineal gland in relation to age, body weight and height. Folia Morphol..

[25] Sigurdardottir L.G., Markt S.C., Sigurdsson S., Aspelund T., Fall K., Schernhammer E., Rider J.R., Launer L., Harris T., Stampfer M.J. (2016). Pineal gland volume assessed by MRI and its correlation with 6-Sulfatoxymelatonin levels among older men. J. Biol. Rhytms.

[26] Sun B., Wang D., Tang Y., Fan L., Lin X., Yu T., Qi H., Li Z., Liu S. (2009). The pineal volume: A three-dimensional volumetric study in healthy young adults using 3.0 T MR data. Int. J. Dev. Neurosci..

[27] Han Q., Li Y., Wang J., Zhao X. (2018). Sex Difference in the Morphology of Pineal Gland in Adults Based on Brain Magnetic Resonance Imaging. J. Craniofac. Surg..

[28] Gheban B.A., Rosca I.A., Crisan M. (2019). The morphological and functional characteristics of the pineal gland. Med. Pharm. Rep..

[29] Mrvelj A., Womble M.D. (2020). Fluoride-Free Diet Stimulates Pineal Growth in Aged Male Rats. Biol. Trace Elem. Res..

[30] Vighl B., Sz A., Debreceni K., Silva M.J.M. (1998). Comparative histology of pineal calcification. Histol. Histopathol..

[31] Doyle A.J., Anderson G.D. (2006). Physiologic calcification of the pineal gland in children on computed tomography: Prevalence, observer reliability and association with choroid plexus calcification. Acad. Radiol..

[32] Golzarian J., Balériaux D., Bank W.O., Matos C., Flament-Durand J. (1993). Pineal cyst: Normal or pathological?. Neuroradiol.

[33] Barboriak D.P., Lee L., Provenzale J.M. (2001). Serial MR imaging of pineal cysts: Implications for natural history and follow-up. Am. J. Roentgenol..

[34] Vaquero J., Martinez R., Escandón J., Bravo G. (1988). Symptomatic glial cysts of the pineal gland. Surg. Neurol..

[35] Pu Y., Mahankali S., Hou J., Li J., Lancaster J.L., Gao J.H., Appelbaum D.E., Fox P.T. (2007). High prevalence of pineal cysts in healthy adults demonstrated by high-resolution, noncontrast brain MR imaging. AJNR Am. J. Neuroradiol..

[36] Fain J.S., Tomlinson F.H., Scheithauer B.W., Parisi J.E., Fletcher G.P., Kelly P.J., Miller G.M. (1994). Symptomatic glial cysts of the pineal gland. J. Neurosurg..

[37] Gurcan M.N., Boucheron L.E., Can A., Madabhushi A., Rajpoot N.M., Yener B. (2009). Histopathological Image Analysis: A Review. IEEE Rev. Biomed. Eng..

